# A low-cost, low-input method establishment for m^6^A MeRIP-seq

**DOI:** 10.1042/BSR20231430

**Published:** 2024-01-09

**Authors:** Wenjuan Xia, Ling Guo, Huapeng Su, Jincheng Li, Jiafeng Lu, Hong Li, Boxian Huang

**Affiliations:** State Key Laboratory of Reproductive Medicine and Offspring Health, Suzhou Affiliated Hospital of Nanjing Medical University, Suzhou Municipal Hospital, Gusu School, Nanjing Medical University, Suzhou, 215002, China

**Keywords:** alternative splicing, anti-m6A antibody, CST, m6A, MeRIP-seq

## Abstract

N6-methyladenosine (m^6^A) is a highly prevalent modification found in mammal mRNA molecules that plays a crucial role in the regulation of cellular function. m^6^A RNA immunoprecipitation sequencing (MeRIP-seq) has been frequently used in transcriptomics research to identify the location of m^6^A. MABE572 (Millipore) is the most widely utilized and efficient anti-m^6^A antibody for MeRIP-seq. However, due to the high dose and price of this antibody, which has also been taken off the market, we discovered that CST's anti-m^6^A antibody can be used instead of MABE572 to map the m^6^A transcriptome. In the present study, we performed different concentrations of the CST anti-m^6^A antibodies with the corresponding initiation RNA of HEK293T cells, 2.5 μg antibody with 1 μg total RNA, 1.25 μg antibody with 0.5 μg total RNA, and 1.25 μg antibody with 0.1 μg total RNA. By comparing the m^6^A peak calling, enriched motifs, alternative splicing events, and nuclear transcripts modified by m^6^A between the CST and Millipore libraries, it was found that the CST library presented similar data to Millipore, even at incredibly low doses. The volume and cost of antibodies are significantly reduced by this refined MeRIP-seq using CST antibody, making it convenient to map future large-scale sample m^6^A methylation.

## Introduction

RNAs are the molecules that perform numerous important functions within cell to control cellular processes such as gene expression, gene post-transcriptional regulation, and gene silencing. More than 170 chemical modifications that post-transcriptionally embellish RNAs have been identified so far [1]. Various modifications can take place on distinct RNA molecules, including messenger RNA (mRNA), transfer RNA (tRNA), and ribosomal RNA (rRNA). N6-methyladenosine (m^6^A) is a widely prevalent and extensively researched mRNA modification in mammals [2,3]. This modification is reversible under the regulation of ‘writers’ and ‘erasers’ [4–6] and can be recognized by ‘readers’ [7–9]. The m^6^A modification plays a critical role in gene post-transcriptional regulation, such as RNA processing [10], splicing [11,12], stability [13], and translation [14]. Most m^6^A modification regulators are expressed in the nucleus, and some of them play an important role in regulating RNA splicing and chromatin state. For instance, HNRNPC, serving as a m^6^A reader, can bind the splicing silencer sequence on pre-mRNA to inhibit splicing events [15]. Another nuclear reader, YTHDC1, targeted at carRNAs modified by METTL3, has been reported to play a role in the decay of carRNAs and chromatin opening [16].

m^6^A RNA immunoprecipitation sequencing (MeRIP-seq) is a widely used technique in transcriptomics research that enables the identification and quantification of RNA modifications [17,18]. Specifically, it enriches RNA fragments modified by m^6^A through binding specific antibodies to the RRACH motif (R = G or A; H = A, C, or U), which can then be sequenced to identify the sites of m^6^A modification. MeRIP-seq makes it possible to map the m^6^A of various organisms, such as zebrafish embryonic development [19], drosophila neuronal development [20], and mouse and human tissues [21,22]. Mapping of m^6^A requires a large amount of total RNA. The required minimum amount of starting RNA is 50 ng with the anti-m^6^A antibody from Millipore (MABE572), revealing the m^6^A landscape of the transcriptome during maternal-to-zygotic transition (MZT). This antibody has been shown to be more sensitive than other commercial antibodies and is widely used for m^6^A profiling in both human and murine tissues. However, the MABE572 antibody requires large quantities, meaning high cost, thus limiting its application to thousands of clinical samples. Moreover, the antibody is basically in stop production, and it has not yet been proven whether the officially produced MABE572I has functional substitutability. There is a need for a low-cost, highly sensitive antibody to detect m^6^A modification.

In the present work, we have tried to evaluate a new commercial antibody from CST (Cell Signaling Technology) by designing different input RNAs that correspond to different antibody dosage levels. We analyze the quantity, abundance, and enriched RNA production of m^6^A detected peaks, as well as RNA splicing events and nuclear transcripts modified by m^6^A. Our findings suggest that a low concentration of CST antibody can obtain similar data compared with the Millipore library when injected with 1 μg RNA samples. Furthermore, by reducing the dose of CST antibodies and corresponding RNA initiation levels, we observed a decrease in the number of peaks. However, there was almost no difference in the amount of enriched RNA. This is particularly significant as it demonstrates that CST antibodies can be used efficiently and cost-effectively in RNA methylation studies, even at lower concentrations.

## Materials and methods

### Cell culture

HEK293T (Human embryonic kidney) cells were cultured in DMEM basic (ThermoFisher, gibco, C11995500BT) with 10% FBS (Gibco, Australia, 10099141), 1% Penicillin/Streptomycin/Amphotericin B Solution (Sterile) (Sangon Biotech, B540733-0010), and 10 μg/ml Ciprofloxacin (YEASEN, 60201ES05) at 37°C with 5% CO_2_. Cell lines have not been authenticated or tested for mycoplasma.

### RNA isolation and purification

Total RNA was extracted from cells using RNAiso Plus (Takara, 9109) following the manufacturer’s instructions. The RNA samples were assessed with 28S/18S ratios > 2 to ensure good quality. To eliminate DNA contamination, Turbo DNase (Invitrogen, AM2239) treatment was implemented by incubating the RNA samples at 37°C for 30 min. Then, the RNA was purified by adding 1/10 volume of 3 M NaAc, 2 μl of glycogen (5 mg/ml, Invitrogen, AM9510), and an equal volume of isopropanol. The mixture was then incubated at −80°C overnight. The RNA was centrifuged for 15 min at full speed, and the RNA pellet was washed twice with 75% ethanol. Finally, the RNA pellet was resolved in RNase-free water. A Qubit RNA HS Assay Kit (Thermo Fisher Scientific, Q32855) was employed to measure the RNA concentration.

### MeRIP

The procedure for MeRIP sequencing was performed according to the published low-input m^6^A-seq protocol with slight modifications [23]. Each group of 15, 1, 0.5, and 0.1 μg total RNA was fragmented into approximately 200 nt-long fragments using RNA Fragmentation Reagents (Thermo Fisher Scientific, AM8740) at 70°C for approximately 5 min in a preheated thermal cycler (Eastwin, ETC821). Then, 2 μl of stop solution was added and mixed (Thermo Fisher Scientific, AM8740). The fragmented RNA was purified by adding 1/10 volume of 3M NaAc, 2 μl of glycogen and, an equal volume of isopropanol, as mentioned above.

Approximately 10 ng of purified RNA was kept as input, and the remaining RNA was prepared for m^6^A-MeRIP. Approximately 30 μl protein G magnetic beads (Thermo Fisher Scientific, 10004D) and 30 μl protein A magnetic beads (Thermo Fisher Scientific, 10002D) were mixed and washed twice with IP buffer (10 mM pH 7.5 Tris-HCl, 150 mM NaCl, and 0.1% IGEPAL CA-630). Then, the mixed beads were resuspended in 200 μl IP buffer. The anti-m^6^A antibodies were added to the resuspended beads, and the mixture was rotated at 4°C overnight. Two anti-m^6^A antibodies were used in the present study. One is 5 µg Millipore anti-m^6^A antibody (Millipore, ABE572). The other is 2.5 µg/1.25 µg CST anti-m^6^A antibody (Cell Signaling Technology, #56593).

The remaining fragmented RNA was added into the pre-cleaning bead-antibody mixture at 4°C for approximately 2 h, followed by being washed twice with IP buffer, washed twice with low-salt IP buffer (10 mM pH 7.5 Tris-HCl, 50 mM NaCl, and 0.1% IGEPAL CA-630), and washed twice with high-salt IP buffer (10 mM pH 7.5 Tris-HCl, 500 mM NaCl, and 0.1% IGEPAL CA-630) for 10 min each at 4°C. After washing, the bound RNA was eluted by competition with 6.7 mM N6-methyladenosine in 200 μl IP buffer (Selleckchem, S3190). Immunoprecipitation RNA was purified with RNeasy MiniElute spin column (Qiagen, 74104), and 14 μl RNase-free H_2_O was used to elute the m^6^A RNA.

### Library preparation

The input RNA (10 ng fragmented RNA) and total IP RNA were used as starting materials to construct libraries with SMARTer Stranded Total RNA-Seq Kit v2-Pico Input Mammalian (Takara-Clontech, 634488), according to the standard protocol. The input RNA underwent 14 PCR cycles, while the m^6^A RNA was subjected to 16 cycles. Sequencing was performed using Illumina NovaSeq 6000 PE150.

### Peak intensity and the calculated overall methylation level

To calculate the methylation intensity of a corresponding region in MeRIP-seq, we use the following formula: (IP FPKM/Input FPKM), where Input FPKM is (counts of mapped fragments × 10^9^) / (Length of peak × Total count of the mapped fragments) and IP FPKM is (Counts of mapped fragments × 10^9^) / (Length of peak × Total count of the mapped m^6^A fragments). To identify the m^6^A methylation level of a gene, we use the following formula: $\sum_{l}^{n}m_{i}l_{i}/L\;(i\in (1,2,\ldots ,n))$, where *m_i_* is the methylation level, *l_i_* is the length of the peak in the gene, *n* is the number of peaks located in the gene, and *L* is the longest transcript length of every gene.

### MeRIP-seq data analysis

The m^6^A sequencing method involved the identification of m^6^A-enriched peaks from all groups using MACS software (2.2.7) [24]. The resulting narrow peak data from all libraries were aligned to the hg19 reference genome from UCSC using the ChIPseeker (1.26.2) package [25]. The m^6^A methylation level of genes was defined as the average value of the score value of corresponding m^6^A peaks. The package ‘Guitar’ [26] was utilized to analyze m^6^A RNA-treated genomic features. The annotation data were visualized by R software (version 4.0.3) and GraphPad Prism 8.0 software. The m^6^A peaks from Millipore and other groups were classified as unique and common based on whether they overlapped or not.

### Quantification of long-read transcripts

To assess the expression of longread transcripts and genes with TPM (Transcripts Per Million) and FPKM (fragments per kilobase million), we used salmon (v0.60.0) to quantify the mapped reads of transcriptome using only GENCODE(GRCh37) annotation or GENCODE annotation augmented with long-read transcripts as reference.

### Motif discovery

All peaks were chosen for m^6^A motif analysis with HOMER (v4.11) in each group [26]. We selected the first ‘RRACH’ motif from the obtained motifs of each group (Millipore, CST I, CST II, and CST III) and recorded the *P* value.

### Unique and overlapping m^6^A peaks

For the intersections of m^6^A peaks among specific groups, we use BEDTools (version 2.29.2) with function ‘intersect’, with default parameter.

### Identification and characterization of AS events

Seven types of alternative splicing (AS) events were identified using SUPPA2 (v2.2.1) in each stage, including skipping exons (SE), alternative 5′ or 3′ splice sites (A5/A3), retained introns (RI), mutually exclusive exons (MX), and alternative first or last exons (AF, AL). Then, the percent spliced in (PSI) values were calculated by SUPPA2 based on the TPM values of transcripts in each sample with psiPerEvent subcommand. AS genes were defined as genes associated with AS events. In each stage, we applied SUPPA2 to calculate the PSI of each AS event using RNA-seq data with the default parameters, and merged long-read transcripts were used as a reference.

### Identification and characterization of paRNA and repeats RNA

Raw sequencing reads were trimmed by Trimmomatic to remove low-quality bases and adapters. The output was first subjected to Trim_galore (http://www.bioinformatics.babraham.ac.uk/projects/trim_galore/) for quality control and trimming adaptors. The quality threshold was set to 20, and the minimum length required for reads after trimming was 30 nt. All the m^6^A-seq and input raw data reads were first aligned against rRNA (hg38, downloaded from UCSC Genome Browser) using bowtie2 (version 2.4.1), with the unmapped reads kept for further analysis. The remaining reads were mapped to GENCODE human hg19 rmsk genome using STAR (version 2.7.8a) with default parameters. Then the results obtained from STAR were transformed into the bam format using BEDTools (version 2.29.2). m^6^A peaks were called using MACS2 (version 2.2.7) with the parameter ‘–nomodel’ separately, and significant peaks with *q* < 0.01 were considered. We constructed the UCSC Genome Browser hg19 annotation file by using ‘makeTxDbFromGFF’ function of the GenomicFeatures R package. The candidate peaks were assigned to the nearest genes by annotatePeak in ChIPseeker. The numerical count of reads in m^6^A peaks was calculated by featureCounts with parameters ‘-p -t exon -g gene_id’.

## Results

### Whole-transcriptome m^6^A profiling in Millipore and CST libraries

**Figure 1:**
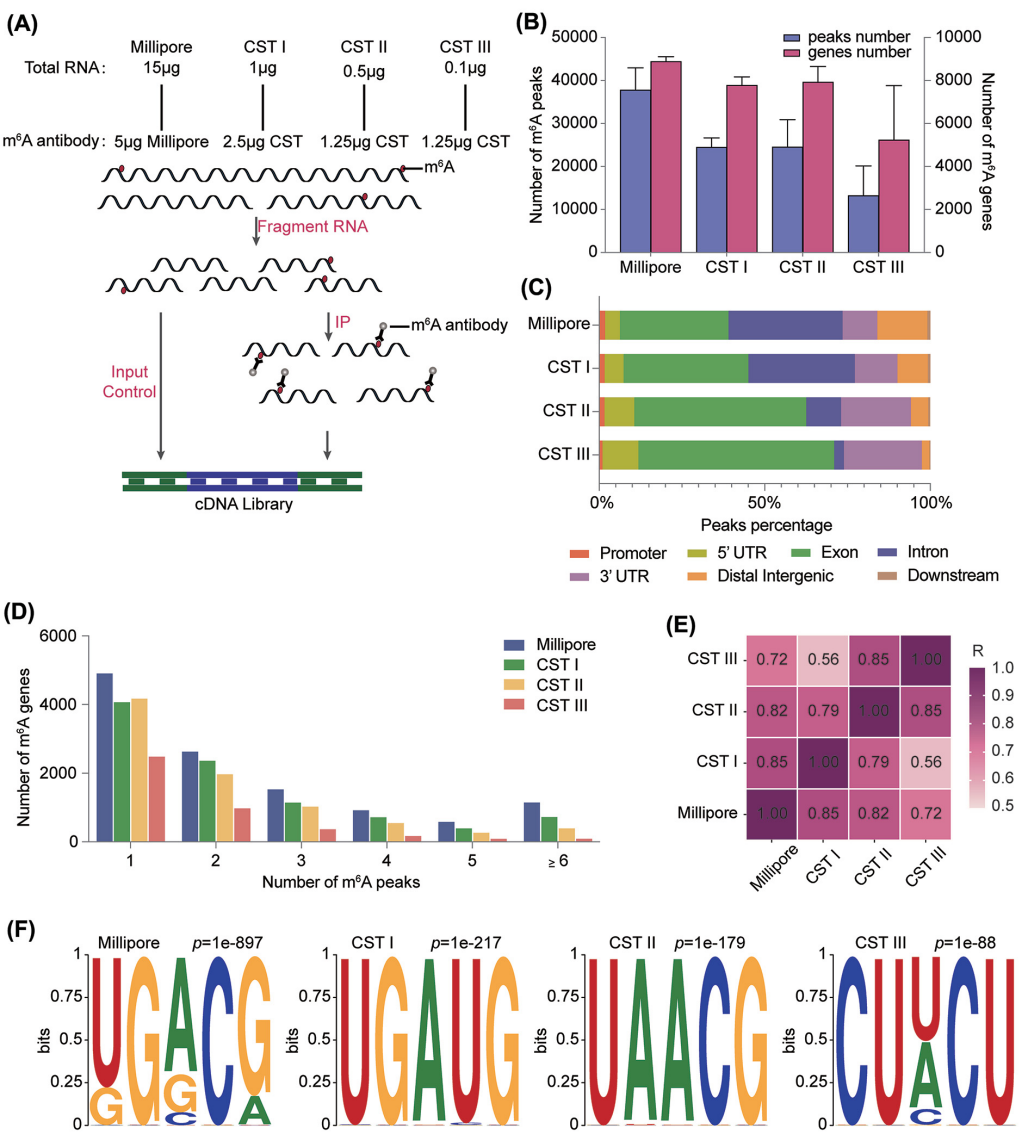
Overview of the m^6^A from different anti-m^6^A libraries in HEK293T cells (**A**) Schematic diagram of the procedure of m^6^A MeRIP and naming of each group with different m^6^A antibodies combined with different starting RNA amounts. (**B**) The total number of m^6^A peaks and m^6^A genes identified by each group. (**C**) The percentages of m^6^A peaks in different transcript segments: promoter, 5′UTR intron, exon, intron, 3′UTR, distal intergenic, and downstream regions. (**D**) The number of m^6^A genes that contain one or more m^6^A peaks (peak = 1, peak = 2, peak = 3, peak = 4, peak = 5, and peak ≥ 6) in each group. (**E**) Heat map of Pearson correlation coefficient between four antibody groups based on common m^6^A genes. (**F**) Top m^6^A motifs identified within m^6^A peaks in each group.

To investigate an optimized protocol for the MeRIP-seq analysis with CST anti-m^6^A antibody, we estimated the performance of different concentrations of the CST anti-m^6^A antibody in relation to the starting amount of RNA from HEK293T cells (CST I: 2.5 μg CST anti-m^6^A antibody with 1 μg total RNA, CST II: 1.25 μg CST anti-m^6^A antibody with 0.5 μg total RNA, CST III: 1.25 μg CST anti-m^6^A antibody with 0.1 μg total RNA) compared to the widely used Millipore anti-m^6^A antibody (Millipore: 5 μg Millipore anti-m^6^A antibody with 15 μg total RNA) (Figure 1A). The optimized MeRIP-seq used extensive high/low salt washing after incubation of the antibody-bead complex and RNA fragments, and then the SMARTer Stranded Total RNA-Seq Kit v2 (Pico Input Mammalian) was employed for library construction as previously reported [23] (Figure 1A). We first compared the number of m^6^A peaks and m^6^A-modified genes among the four groups. Millipore captured an obviously larger number of m^6^A peaks (30957) and genes (8314) compared with CST. In the other three groups, CST I identified the most m^6^A peaks (23188) and m^6^A genes (7108), while CST II detected slightly fewer m^6^A peaks (14842) and genes (6919), and CST III showed significant decreases in both (Figure 1B). In all groups, the m^6^A peaks were most abundant near the stop codon, 3′UTR, and internal coding sequence (CDS) (Supplementary Figure S1). This was similar to what other studies had found [27]. Then, we analyzed the distribution of differential m^6^A peaks in the transcriptome. The distribution of m^6^A peaks in the genome was quite similar between Millipore and CST I, while in CST II and CST III, the m^6^A peaks were enriched more in the 5′UTR, exon, and 3′UTR and less in the intron (Figure 1C). On average, there are 3-5 m^6^A peaks on each m^6^A-modified mRNA. Based on our analysis, most of the m^6^A genes exhibited one or two m^6^A peaks, and Millipore showed obvious advantages in the m^6^A gene number among all m^6^A peak groups (Figure 1D). The difference in the total number of genes between CST I and Millipore can be explained by whether or not m^6^A peak sites were found (Figure 1B,D). CST II exhibited a similar number of genes containing 1-3 m^6^A peaks as CST I. However, in the CST III group, the number of m^6^A genes identified was very small, whatever the group of m^6^A peaks (Figure 1D). As expected, samples clustered well according to m^6^A genes in all four groups. Additionally, both CST I and CST II were highly correlated with Millipore (Figure 1E). Transcriptome-wide m^6^A site mapping revealed the typical consensus sequence RRACH (R = G or A; H = A, C, or U). The top consensus motif in CST I and CST II, conforming to the m^6^A sequence feature, tended to be close to the motif enriched in Millipore (Figure 1F), while CST III was inconsistent. So we will no longer analyze CST III in the future.

These results indicated that the m^6^A peaks and genes identified in the four groups were highly trusted. Although Millipore has been shown to have great advantages with regular amounts of RNA, CST I and CST II appear to be reliable substitutes for MeRIP-seq with low input RNA.

### m^6^A enrichment genes in CST II approach to Millipore

**Figure 2:**
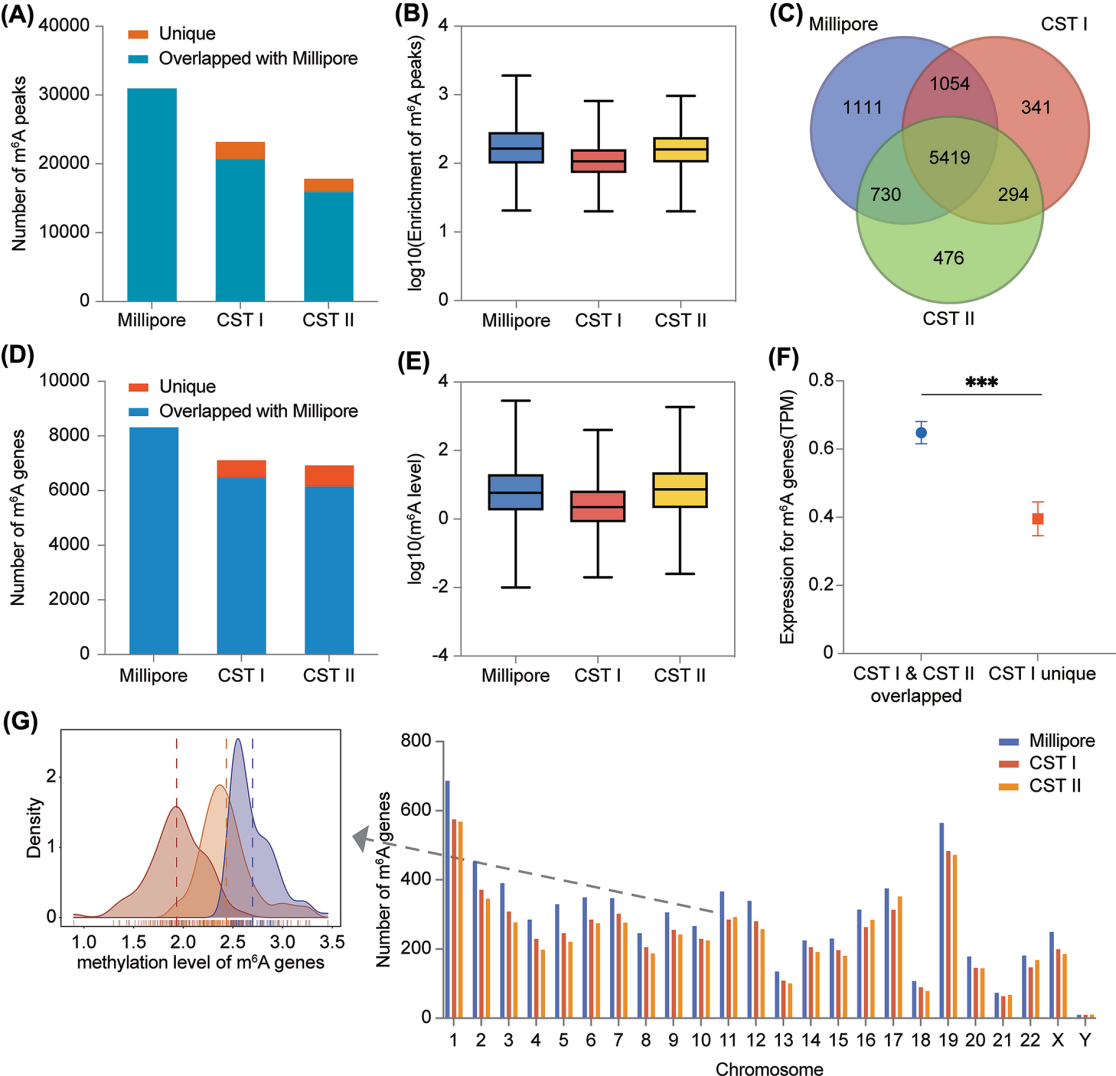
Enrichment of m^6^A peaks and genes in Millipore, CST I and CST II (**A**) The total m^6^A peak number of Millipore, overlapped, and unique m^6^A peaks identified by CST I and CST II when compared to Millipore. (**B**) Relative enrichment of total m^6^A peaks in three groups. (**C**) Venn diagram showing the overlap of m^6^A genes in three groups. (**D**) Total m^6^A gene number of Millipore, overlapped and unique m^6^A genes identified by CST I and CST II when compared with Millipore. (**E**) The m^6^A methylation levels of total m^6^A genes in three groups. (**F**) The average RNA expression level of the unique m^6^A genes identified in CST I was significantly lower than that of m^6^A genes overlapped with CST II, ***P < 1 × 10^−4^. (**G**) Left: Density plot of overall m^6^A level of 100 randomly selected genes on random chromosomes in three groups. Right: Distribution of m^6^A genes on chromosomes in three groups.

To compare the consistency between CST and millipore antibodies, we analyzed the obtained sequencing data from various aspects. First, we detected m^6^A peaks and m^6^A genes, those exhibited in CST I and CST II were highly overlapped when compared with Millipore (Figure 2A,D). Although CST I detected more m^6^A peaks (Figure 2A) than CST II, a similar fraction of unique m^6^A peaks in CST I and CST II was observed – approximately 20% of themselves (Supplementary Figure S2A). And CST II identified an even higher enrichment of total m^6^A peaks than CST I (Figure 2B). To further investigate the similarity and difference of m^6^A-modified genes in the three groups, a Venn diagram was shown (Figure 2C). We found that approximately 75% of m^6^A genes were common to all three groups. Compared with CST I, CST II showed greater m^6^A enrichment in both the common peaks and the peaks that overlapped with Millipore (Supplementary Figure S2B and S2C).

In terms of genes overlapped with Millipore, the number of unique m^6^A-modified genes in CST II was slightly higher than that in CST I (Figure 2D and Supplementary Figure S2D). In addition, we evaluated the m^6^A levels of overall genes with m^6^A modification and the overlapped 5419 m^6^A genes in the three groups and found that CST II was more similar to Millipore (Figure 2E and Supplementary Figure S2E). We focused more on the correlation of common m^6^A genes among the three groups, and the results indicated that the majority of the m^6^A levels of every single m^6^A gene in CST II were closer to Millipore than in CST I (Supplementary Figure S2F). This may explain the reason why CST II targeted fewer m^6^A genes but identified a higher m^6^A level than CST I (Figure 2A,B). Compared witg CST I, the m^6^A levels of genes that overlapped with Millipore in CST II were much more similar to those of Millipore (Supplementary Figure S2G). Then, we grouped m^6^A genes by m^6^A levels. The top 30% of genes with the highest m^6^A methylation level were defined as high m^6^A level genes, the bottle 30% of genes were defined as low m^6^A level genes, and the rest 40% were defined as middle m^6^A level genes. Similar performance was also observed in m^6^A genes that overlapped with Millipore in both CST I and CST II, with CST II showing characteristics similar to those of Millipore (Supplementary Figure S2H). We next calculated the average expression level of the overlapped m^6^A genes between CST I and CST II with unique m^6^A genes in CST I. The results showed that the expression of unique m^6^A genes in CST I was relatively low, and these genes may not arouse close attention in reality (Figure 2F). Furthermore, we also calculated the number of m^6^A genes distributed on each chromosome in the three groups (Figure 2G, right). Briefly, Millipore identified the highest m^6^A genes on all chromosomes, while CST I detected more m^6^A genes than CST II, except on chromosome 11, 16, 17, 21, and 22. In addition, we randomly extracted 100 m^6^A genes from each of the three groups, and the density plot consistently showed a stronger correlation between the m^6^A levels of CST II and Millipore (Figure 2G, left).

In summary, CST I may have a significant advantage in the number of m^6^A peaks and genes, but CST II tends to be closer to Millipore at the m^6^A level and density of genes.

### CST I captures greater consistent alternative splicing events than CST II

**Figure 3:**
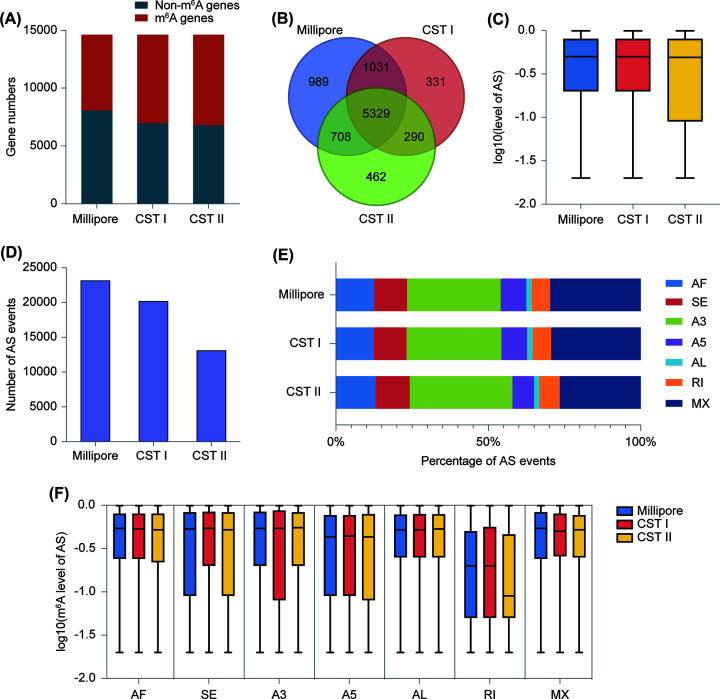
Characterization of alternative splicing of m^6^A genes in CST I, CST II and Millipore (**A**) The fraction of non-m^6^A and m^6^A modification in alternative splicing genes in three antibody groups. (**B**) Venn diagram showing the overlap of alternative splicing genes with m^6^A modification. (**C**) The overall level of alternative splicing in three groups. (**D**) The number of alternative splicing events with m^6^A modification in three groups. (**E**) The percentage of alternative first exon (AF), skipping exon (SE), alternative 3′splice site (A3), alternative 5′splice site (A5), alternative last exon (AL), retained intron (RI), and mutually exclusive exons (MX) with m^6^A modification in three groups. (**F**) The level of each alternative splicing type in three groups.

m^6^A is a crucial modification involved in the processing of pre-mRNA. The m^6^A reader YTHDC1 has been directly proven to regulate the splicing of mRNA [28]. We investigated the correlation between m^6^A methylation and alternative splicing (AS) events using three anti-m^6^A groups: CST I, CST II, and Millipore. All three groups displayed similar total mRNA AS events using SUPPA2 [29] (Supplementary Figure S3A). However, we found that m^6^A modified roughly half of the spliced genes. For example, in the CST I group, 7,624 out of 14,605 AS genes underwent m^6^A modification (Figure 3A), highlighting the importance of m^6^A in splicing events. Moreover, the m^6^A-modified spliced genes in both CST I and CST II had a lot in common with those in Millipore (Figure 3B). We observed a concordance probability in m^6^A gene AS events among the three groups, with no significant difference (Figure 3C). Notably, Millipore exhibited a significant advantage in AS events with m^6^A modification, reaching 23,140 (Figure 3D). Although the number of AS events in CST I (20,173) was relatively smaller than in Millipore, the distribution of the seven AS types and the m^6^A methylation levels of the spliced genes in CST I were consistent with those of Millipore (Figure 3E,F), and this concordance was greater than that of CST II. Conversely, the AS event count of m^6^A genes in CST II was much smaller than in Millipore (Figure 3D), and the distribution of total mRNA AS was similar among all three antibody groups (Supplementary Figure S3B). Additionally, to further explore the role of m^6^A modification level in AS events, we grouped m^6^A genes based on their m^6^A levels, as mentioned previously. Similarly, CST I exhibited greater concordance with Millipore in the three m^6^A level groups (Supplementary Figure S3C). These results suggest that genes modified by m^6^A in CST I are more similar to those of Millipore in terms of the number of splicing events, splicing probability, and distribution of splicing types. Therefore, the use of CST I antibodies can, to some extent, replace Millipore antibodies.

### CST II marks greater consistent m^6^A -modified nuclear transcripts than CST I

**Figure 4:**
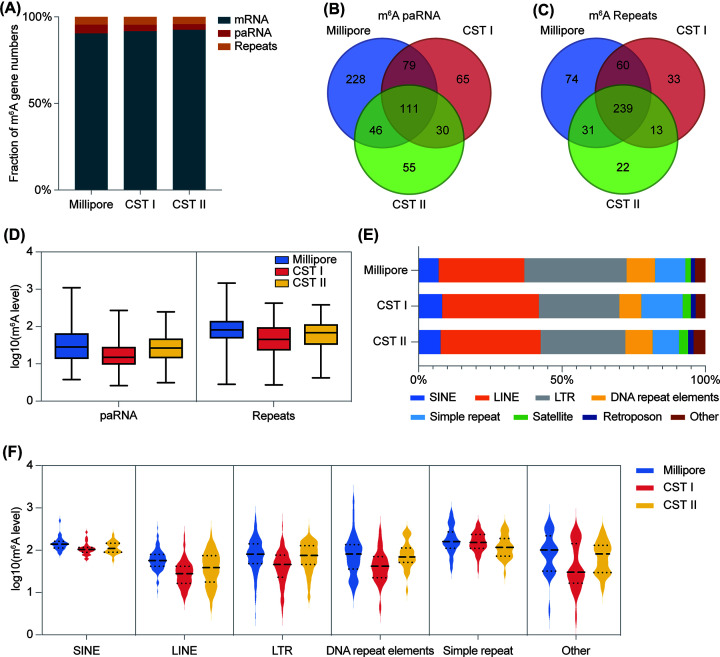
Characterization of paRNA and Repeats RNA m^6^A-modified transcripts in the nucleus of CST I and CST II (**A**) The fraction of mRNA, paRNA and Repeats with m^6^A modification in three groups. (**B**) Venn diagram showing the overlap of m^6^A paRNA in three groups. (**C**) Venn diagram showing the overlap of m^6^A Repeats in three groups. (**D**) The overall methylation levels of paRNA and Repeats in three groups. (**E**) The percentages of different Repeat types with m^6^A modification: SINE, LINE, LTR, DNA repeat elements, simple repeat, Satellite, Retroposon and other types. (**F**) The methylation levels of different Repeat types with m^6^A modification: SINE, LINE, LTR, DNA repeat elements, simple repeat, Satellite, Retroposon and other types in three groups.

Chromatin-associated regulatory RNAs (carRNAs) are RNAs that associate with chromatin directly or indirectly. They have been found to be involved in gene and transcriptional regulation through multiple mechanisms and have important roles in different types of cancer [30]. Approximately 15–30% of carRNAs contain m^6^A in mESCs. m^6^A facilitates the degradation of carRNAs by regulating their stability and chromatin state [16,31]. In addition to mRNA, we also detected carRNAs in HEK293T cells, including promoter-associated RNA (paRNA) and RNA transcribed from transposable elements (repeats RNA). In the three groups, the overwhelming majority of the m^6^A-modified transcripts were mRNA, and promoter associated RNA (paRNA) as well as repeats only constituted a small percentage, approximately 8% (Figure 4A). Millipore still showed a superior ability to detect paRNA and Repeats, while CST II was only a little inferior to CST I (Figure 4B,C). The Venn diagram shows a great diversity of paRNA, but repeats were largely overlapped among the three groups. We further estimated the m^6^A level of paRNA and Repeats in each group. It turned out that the m^6^A levels of paRNA and Repeats in CST II were closer than those in CST I to those in Millipore (Figure 4D). Whatever the paRNA and Repeats in common across the three groups, and whatever the paRNA and repeats in CST I or CST II overlapped with Millipore, the results were also observed (Supplementary Figure S4A and S4B). Then, we analyzed the distribution of different repeat types with m^6^A modifications among the three groups. Both CST I and CST II enriched more LINE, CST I targeted more simple repeats than CST II (Figure 4E). But other than this, the percentage distribution of CST II resembled that of Millipore in general (Figure 4E). We further investigated the m^6^A level of each repeat type and found that the m^6^A levels of CST II were higher than CST I except for simple repeat (Figure 4F). These results indicate that CST II is more similar to Millipore in terms of the numbers and m^6^A levels of different carRNAs.

## Discussion

MABE572, which was Millipore’s most commonly used anti-m^6^A antibody in MeRIP-seq to detect m^6^A modifications, has stopped being made. In the present study, we developed an anti-m^6^A antibody from CST for m^6^A mapping by comparing the transcripts, alternative splicing events, and the carRNAs in the nucleus that m^6^A marked, with the data from Millipore. Importantly, the m^6^A-marked peaks and genes of CST are the same as those of Millipore with less antibody concentration and less RNA. This saves both money and samples.

Each mRNA contains three m^6^A sites, but most m^6^A-marked mRNAs have only one m^6^A peak. Our results confirmed this observation. Whatever the three libraries of CST or Millipore, the number of genes with one m^6^A site was the highest. We found that even though CST II had fewer total m^6^A peaks than CST I, the number of m^6^A genes with a single m^6^A peak was higher in CST II than in CST I. This meant that the total number of m^6^A-modified genes was the same for both. We speculate that because CST is a high-titer antibody, it can lead to the superabundant binding of antibodies to protein A/G. The many effective binding sites for m^6^A of crowded antibodies may be embedded, making it very difficult for RNA containing m^6^A to approach and incorporate with the binding site of antibodies. In contrast, a low concentration of CST creates a suitable proportion of antibody-bead complexes to capture m^6^A RNA fragments under optimized conditions. This may explain why CST II was able to identify almost an equal amount of enriched RNA as CST I by reducing the use of CST antibodies and corresponding RNA initiation levels. When there is an excessive amount of CST antibody that can't effectively bind modification sites in CST III, the ratio of antibodies to input RNA can affect the effectiveness of IP.

Previous research has proposed that m^6^A-dependent RNA modification is important for alternative splicing regulation [32,33]. Zhao et al. showed the function of FTO, a m^6^A ‘eraser’, in the mediation of mRNA splicing and gene expression with transcriptome analyses of m^6^A-seq [12]. The exon junction complex (EJC) is recruited by spliceosome to inhibit METTL3-mediated m^6^A modification [34]. We also examined the efficiency of the CST antibody when it comes to alternative splicing with our MeRIP-seq. We observed that m^6^A-modified MX events were almost the most common (Figure 3E), but they were the least common in total mRNA (Figure 3B) out of the seven different types of alternative splicing. Mutually exclusive splicing is a specific type of alternative splicing in which only one of two or more reduplicated exons is spliced into the mature mRNA isoform. MXs on *Dscam* contribute to 38016 isoforms in Drosophila melanogaster [35]. MXs have been reported to be associated with protein modification functions [36]. The various isoforms produced maintain diversity in biological functions, and transcripts produced by different MXs exhibit tissue specificity. In both CST and Millipore libraries, there is an abundance of m^6^A on MX events, indicating that this is not a random occurrence. This suggests that m^6^A modification may influence protein functions by modifying mutually exclusive splicing, which may be related to the development of diseases and play a role in biological evolution.

This study has some limitations. We did not carry out MeRIP-seq in more types of cells or tissues. Subsequent studies of more abundant sample types and larger sample sizes are needed to demonstrate our findings. On the other hand, whether CST antibodies have a tissue preference, other commercial antibodies and tissues from different species should also be included in the study for comparison.

## Conclusion

In conclusion, we utilized three distinct methodologies, each utilizing varying concentrations of the CST antibody with initial total RNA from HEK293T cell lines, to cater to the diverse information requirements captured by MeRIP-seq. In terms of comparing m^6^A peak calling, m^6^A-genes, and transcripts methylated in the nucleus with Millipore libraries, CST I exhibits superior performance in splicing events. Conversely, CST II proves to be more effective when assessing the total number of m^6^A-genes and carRNAs. Both libraries ensure data correspondence with Millipore, even at low concentrations of m^6^A-antibody and with minimal input RNA. Our study not only conserves cost resources but also facilitates the mapping of m^6^A methylation in large-scale future samples.

## Supplementary Material

Supplementary Figures S1-S4

## Data Availability

All data are available from the corresponding authors upon reasonable request.

## Abbreviations

AS: alternative splicing
carRNA: chromatin-associated regulatory RNA
CDS: coding sequence
EJC: exon junction complex
m^6^A: N6-methyladenosine
MeRIP-seq: m^6^A RNA immunoprecipitation sequencing
paRNA: promoter-associated RNA
PSI: percent spliced in

## References

[1] Boccaletto P., Stefaniak F., Ray A., Cappannini A., Mukherjee S., Purta E. et al. (2022) MODOMICS: a database of RNA modification pathways. 2021 update. Nucleic Acids Res. 50, D231–D235 10.1093/nar/gkab1083 34893873 PMC8728126

[2] Tuck M.T. (1992) The formation of internal 6-methyladenine residues in eucaryotic messenger RNA. Int. J. Biochem. 24, 379–386 10.1016/0020-711X(92)90028-Y 1551452

[3] Zhao B.S., Roundtree I.A. and He C. (2017) Post-transcriptional gene regulation by mRNA modifications. Nat. Rev. Mol. Cell Biol. 18, 31–42 10.1038/nrm.2016.132 27808276 PMC5167638

[4] Jia G., Fu Y., Zhao X., Dai Q., Zheng G., Yang Y. et al. (2011) N6-methyladenosine in nuclear RNA is a major substrate of the obesity-associated FTO. Nat. Chem. Biol. 7, 885–887 10.1038/nchembio.687 22002720 PMC3218240

[5] Liu J., Yue Y., Han D., Wang X., Fu Y., Zhang L. et al. (2014) A METTL3-METTL14 complex mediates mammalian nuclear RNA N6-adenosine methylation. Nat. Chem. Biol. 10, 93–95 10.1038/nchembio.1432 24316715 PMC3911877

[6] Zheng G., Dahl J.A., Niu Y., Fedorcsak P., Huang C.M., Li C.J. et al. (2013) ALKBH5 is a mammalian RNA demethylase that impacts RNA metabolism and mouse fertility. Mol. Cell. 49, 18–29 10.1016/j.molcel.2012.10.015 23177736 PMC3646334

[7] Alarcon C.R., Goodarzi H., Lee H., Liu X., Tavazoie S. and Tavazoie S.F. (2015) HNRNPA2B1 is a mediator of m(6)A-dependent nuclear RNA processing events. Cell 162, 1299–1308 10.1016/j.cell.2015.08.011 26321680 PMC4673968

[8] Huang H., Weng H., Sun W., Qin X., Shi H., Wu H. et al. (2018) Recognition of RNA N(6)-methyladenosine by IGF2BP proteins enhances mRNA stability and translation. Nat. Cell Biol. 20, 285–295 10.1038/s41556-018-0045-z 29476152 PMC5826585

[9] Shi H., Wang X., Lu Z., Zhao B.S., Ma H., Hsu P.J. et al. (2017) YTHDF3 facilitates translation and decay of N(6)-methyladenosine-modified RNA. Cell Res. 27, 315–328 10.1038/cr.2017.15 28106072 PMC5339834

[10] Alarcon C.R., Lee H., Goodarzi H., Halberg N. and Tavazoie S.F. (2015) N6-methyladenosine marks primary microRNAs for processing. Nature 519, 482–485 10.1038/nature14281 25799998 PMC4475635

[11] He P.C., Wei J., Dou X., Harada B.T., Zhang Z., Ge R. et al. (2023) Exon architecture controls mRNA m(6)A suppression and gene expression. Science 379, 677–682 10.1126/science.abj9090 36705538 PMC9990141

[12] Zhao X., Yang Y., Sun B.F., Shi Y., Yang X., Xiao W. et al. (2014) FTO-dependent demethylation of N6-methyladenosine regulates mRNA splicing and is required for adipogenesis. Cell Res. 24, 1403–1419 10.1038/cr.2014.151 25412662 PMC4260349

[13] Wang X., Lu Z., Gomez A., Hon G.C., Yue Y., Han D. et al. (2014) N6-methyladenosine-dependent regulation of messenger RNA stability. Nature 505, 117–120 10.1038/nature12730 24284625 PMC3877715

[14] Slobodin B., Han R., Calderone V., Vrielink J., Loayza-Puch F., Elkon R. et al. (2017) Transcription impacts the efficiency of mRNA translation via co-transcriptional N6-adenosine methylation. Cell 169, 326e12–337e12 10.1016/j.cell.2017.03.031 28388414 PMC5388891

[15] Huang X.T., Li J.H., Zhu X.X., Huang C.S., Gao Z.X., Xu Q.C. et al. (2021) HNRNPC impedes m(6)A-dependent anti-metastatic alternative splicing events in pancreatic ductal adenocarcinoma. Cancer Lett. 518, 196–206 10.1016/j.canlet.2021.07.016 34271104

[16] Liu J., Dou X., Chen C., Chen C., Liu C., Xu M.M. et al. (2020) N (6)-methyladenosine of chromosome-associated regulatory RNA regulates chromatin state and transcription. Science 367, 580–586 10.1126/science.aay6018 31949099 PMC7213019

[17] Dominissini D., Moshitch-Moshkovitz S., Salmon-Divon M., Amariglio N. and Rechavi G. (2013) Transcriptome-wide mapping of N(6)-methyladenosine by m(6)A-seq based on immunocapturing and massively parallel sequencing. Nat. Protoc. 8, 176–189 10.1038/nprot.2012.148 23288318

[18] Dominissini D., Moshitch-Moshkovitz S., Schwartz S., Salmon-Divon M., Ungar L., Osenberg S. et al. (2012) Topology of the human and mouse m6A RNA methylomes revealed by m6A-seq. Nature 485, 201–206 10.1038/nature11112 22575960

[19] Zhao B.S., Wang X., Beadell A.V., Lu Z., Shi H., Kuuspalu A. et al. (2017) m(6)A-dependent maternal mRNA clearance facilitates zebrafish maternal-to-zygotic transition. Nature 542, 475–478 10.1038/nature21355 28192787 PMC5323276

[20] Lence T., Akhtar J., Bayer M., Schmid K., Spindler L., Ho C.H. et al. (2016) m(6)A modulates neuronal functions and sex determination in Drosophila. Nature 540, 242–247 10.1038/nature20568 27919077

[21] Xiao S., Cao S., Huang Q., Xia L., Deng M., Yang M. et al. (2019) The RNA N(6)-methyladenosine modification landscape of human fetal tissues. Nat. Cell Biol. 21, 651–661 10.1038/s41556-019-0315-4 31036937

[22] Liu J., Li K., Cai J., Zhang M., Zhang X., Xiong X. et al. (2020) Landscape and regulation of m(6)A and m(6)Am methylome across human and mouse tissues. Mol. Cell. 77, 426e6–440e6 10.1016/j.molcel.2019.09.032 31676230

[23] Zeng Y., Wang S., Gao S., Soares F., Ahmed M., Guo H. et al. (2018) Refined RIP-seq protocol for epitranscriptome analysis with low input materials. PLoS Biol. 16, e2006092 10.1371/journal.pbio.2006092 30212448 PMC6136692

[24] Liu T. (2014) Use model-based Analysis of ChIP-Seq (MACS) to analyze short reads generated by sequencing protein-DNA interactions in embryonic stem cells. Methods Mol. Biol. 1150, 81–95 10.1007/978-1-4939-0512-6_4 24743991

[25] Yu G., Wang L.G. and He Q.Y. (2015) ChIPseeker: an R/Bioconductor package for ChIP peak annotation, comparison and visualization. Bioinformatics 31, 2382–2383 10.1093/bioinformatics/btv145 25765347

[26] Zhou Y., Zhou B., Pache L., Chang M., Khodabakhshi A.H., Tanaseichuk O. et al. (2019) Metascape provides a biologist-oriented resource for the analysis of systems-level datasets. Nat. Commun. 10, 1523 10.1038/s41467-019-09234-6 30944313 PMC6447622

[27] Meyer K.D., Saletore Y., Zumbo P., Elemento O., Mason C.E. and Jaffrey S.R. (2012) Comprehensive analysis of mRNA methylation reveals enrichment in 3' UTRs and near stop codons. Cell 149, 1635–1646 10.1016/j.cell.2012.05.003 22608085 PMC3383396

[28] Xiao W., Adhikari S., Dahal U., Chen Y.S., Hao Y.J., Sun B.F. et al. (2016) Nuclear m(6)A Reader YTHDC1 Regulates mRNA Splicing. Mol. Cell. 61, 507–519 10.1016/j.molcel.2016.01.012 26876937

[29] Trincado J.L., Entizne J.C., Hysenaj G., Singh B., Skalic M., Elliott D.J. et al. (2018) SUPPA2: fast, accurate, and uncertainty-aware differential splicing analysis across multiple conditions. Genome Biol. 19, 40 10.1186/s13059-018-1417-1 29571299 PMC5866513

[30] Tang J., Wang X., Xiao D., Liu S. and Tao Y. (2023) The chromatin-associated RNAs in gene regulation and cancer. Mol. Cancer 22, 27 10.1186/s12943-023-01724-y 36750826 PMC9903551

[31] Wei J., Yu X., Yang L., Liu X., Gao B., Huang B. et al. (2022) FTO mediates LINE1 m(6)A demethylation and chromatin regulation in mESCs and mouse development. Science 376, 968–973 10.1126/science.abe9582 35511947 PMC9746489

[32] Mendel M., Delaney K., Pandey R.R., Chen K.M., Wenda J.M., Vågbø C.B. et al. (2021) Splice site m(6)A methylation prevents binding of U2AF35 to inhibit RNA splicing. Cell 184, 3125.e25–3142.e25 10.1016/j.cell.2021.03.062 33930289 PMC8208822

[33] Liu N., Dai Q., Zheng G., He C., Parisien M. and Pan T. (2015) N(6)-methyladenosine-dependent RNA structural switches regulate RNA-protein interactions. Nature 518, 560–564 10.1038/nature14234 25719671 PMC4355918

[34] Yang X., Triboulet R., Liu Q., Sendinc E. and Gregory R.I. (2022) Exon junction complex shapes the m(6)A epitranscriptome. Nat. Commun. 13, 7904 10.1038/s41467-022-35643-1 36550132 PMC9780246

[35] Graveley B.R. (2005) Mutually exclusive splicing of the insect Dscam pre-mRNA directed by competing intronic RNA secondary structures. Cell 123, 65–73 10.1016/j.cell.2005.07.028 16213213 PMC2366815

[36] Letunic I., Copley R.R. and Bork P. (2002) Common exon duplication in animals and its role in alternative splicing. Hum. Mol. Genet. 11, 1561–1567 10.1093/hmg/11.13.1561 12045209

