# Supplementary material for: A low-cost, low-input method establishment for m6A MeRIP-seq
Source: Biosci Rep. 2024 Jan 9;44(1):BSR20231430. doi: 10.1042/BSR20231430 (PMC10776898; doi:10.1042/BSR20231430)
Supplement: Supplementary Figures S1-S4 [file BSR-2023-1430_supp.pdf]

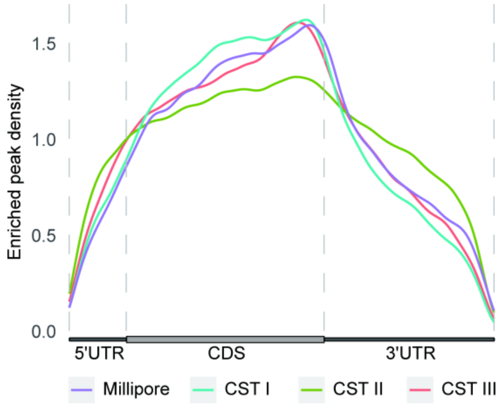

Supplementary Figure 1. Distribution of the enriched m^{6}A peaks in each group.

Distribution of the enriched m^{6}A peaks in all four antibody groups.

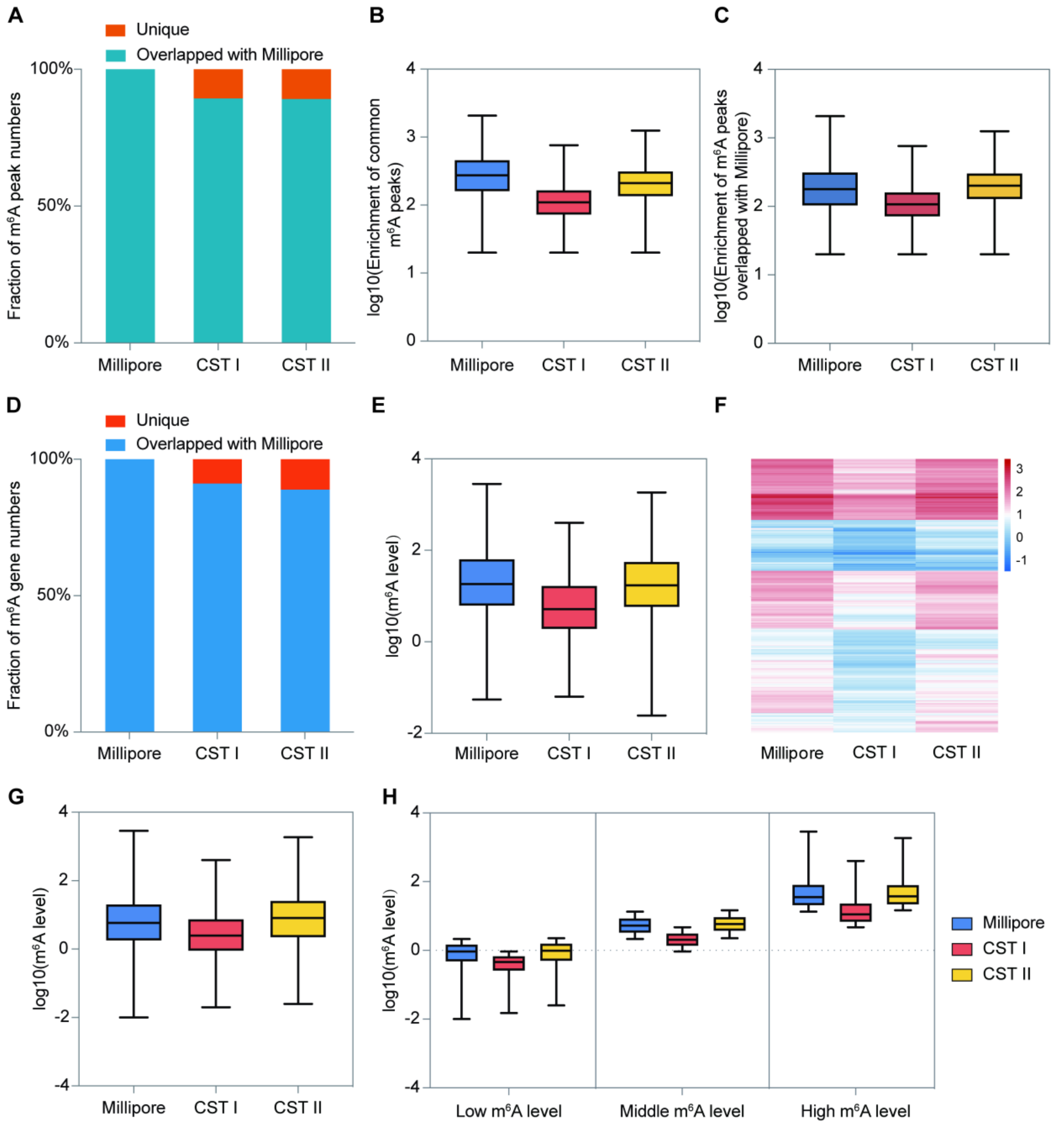

Supplementary Figure 2. Enrichment of m^{6}A peaks and genes with Millipore in CST I and CST II.

(A) The fraction of m^{6}A peak numbers identified by CST I and CST II, “unique” and “overlapped with Millipore” are compared to Millipore. (B) Relative enrichment of common m^{6}A peaks across three groups. (C) Relative enrichment of m^{6}A peaks in Millipore and “overlapped with Millipore” in CST I and CST II. (D) The fraction of overlapped and unique m^{6}A gene numbers compared to Millipore identified by CST I and CST II. (E) The overall methylation levels of common m^{6}A genes across three groups. (F) Heatmap showing the m^{6}A levels of the common gene across the three groups. (G) The

overall methylation levels of m^6A genes in Millipore and m^6A genes overlapped with Millipore in CST I, and CST II. (H) The overall methylation levels of low, middle and high m^6A level genes in three groups.

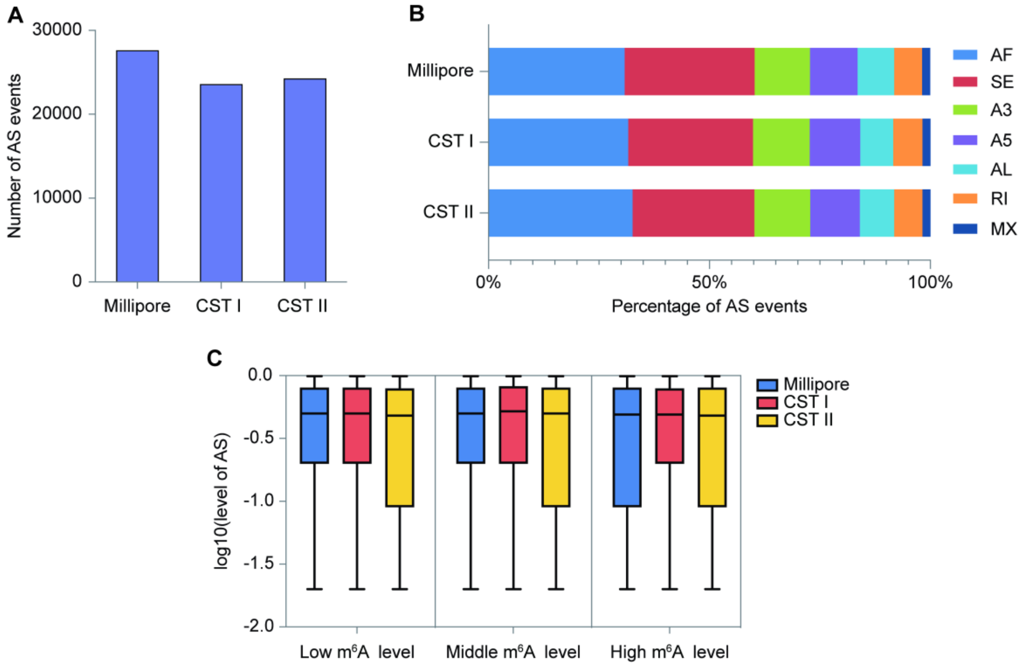

Supplementary Figure 3. Characterization of alternative splicing of mRNA in CST I, CSTII and Millipore.

(A) The number of all alternative splicing events in three groups. (B) The percentage of alternative first exon (AF), skipping exon (SE), alternative 3'splice site (A3), alternative 5'splice site (A5), alternative last exon (AL), retained intron (RI), and mutually exclusive exons (MX) in three groups. (C) The overall level of alternative splicing at low, middle and high m^6A level genes.

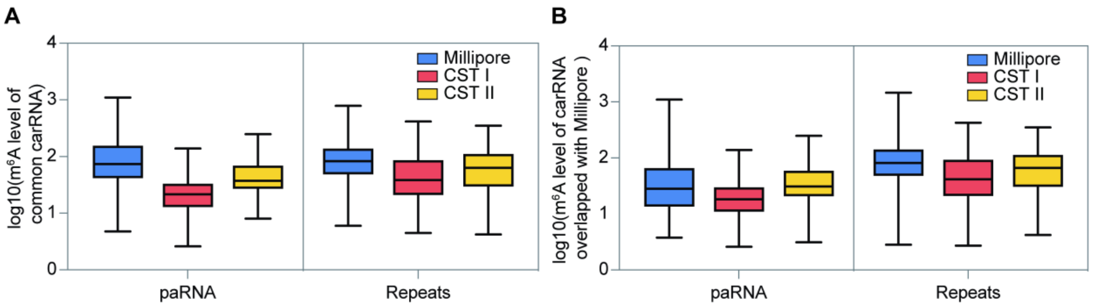

Supplementary Figure 4. Characterization of paRNA and Repeats RNA m^{6}A levels in the nucleus of CST I and CST II.

(A) The overall m^{6}A levels of paRNA and Repeats in common in three groups.

(B) The overall m^{6}A levels of paRNA and Repeats in Millipore, CST I or CST II overlapped with Millipore.
